# Multilayered LDH/Microcapsule Smart Epoxy Coating for Corrosion Protection

**DOI:** 10.1021/acsomega.2c06406

**Published:** 2023-08-18

**Authors:** Norhan Ashraf Ismail, R. A. Shakoor, Noora Al-Qahtani, Ramazan Kahraman

**Affiliations:** † Center for Advanced Materials, Qatar University, Doha 2713, Qatar; ‡ Department of Chemical Engineering, College of Engineering, Qatar University, Doha 2713, Qatar; § Department of Mechanical and Industrial Engineering, College of Engineering, Qatar University, Doha 2713, Qatar

## Abstract

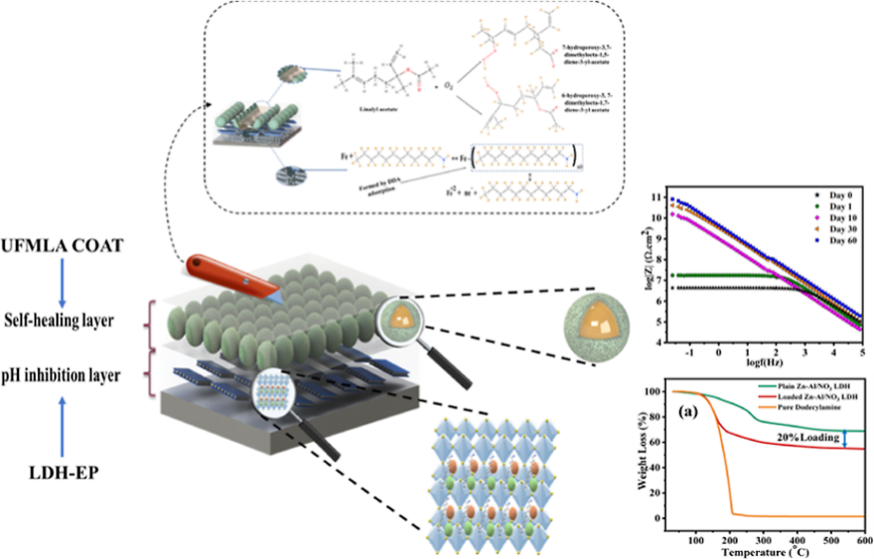

A multilayered smart epoxy coating for corrosion prevention of carbon steel was developed and characterized. Toward this direction, as a first step, zinc-aluminum nitrate-layered double hydroxide (Zn/Al LDH) was synthesized using the hydrothermal crystallization technique and then loaded with dodecylamine (DOD), which was used as an inhibitor (pH-sensitive). Similarly, the synthesis of the urea-formaldehyde microcapsules (UFMCs) has been carried out using the in-situ polymerization method, and then the microcapsules (LAUFCs) were encapsulated with linalyl acetate (LA) as a self-healing agent. Finally, the loaded Zn/Al LDH (3 wt %) and modified LAUFCs (5 wt %) were reinforced into an epoxy matrix to develop a double-layer coating (DL-EP). For an exact comparison, pre-layer epoxy coatings comprising 3 wt % of the loaded Zn/Al LDH (referred to as LDH-EP), top-layer epoxy coatings comprising 5 wt % linalyl acetate urea-formaldehyde microcapsules (referred to as UFMLA COAT), and a blank epoxy coating (reference coating) were also developed. The developed epoxy coatings were characterized using various techniques such as XRD, XPS, BET, TGA, FTIR, EIS, etc. Electrochemical tests performed on the synthesized coatings indicate that the DL-EP demonstrates improved self-healing properties compared to LDH-EP and UFMLA COAT.

## 1 Introduction

Environmental changes always affect materials’ characteristics and interpretation. Corrosion is one of the most prevalent and harmful processes that harm materials. One of the most critical global problems, particularly in the industrial sector, is corrosion, which frequently leads to equipment damage and production interruption. Of several corrosion protection techniques, protective coating is one of the most effective and has attracted many researchers’ attention, especially in the last decade.^1−5^ These coatings are applied on the metal surface to act as a physical barrier against corrosive media. A new class of coatings known as “smart coatings” has characteristics that enable them to sense changes in surroundings and react appropriately to any external stimuli to heal any damage.^6^ The penetration of corrosive substances into the metal is often caused by external stimuli such as mechanical damage or scratches, which results in substance deterioration.^7^ However, smart coatings have a decent ability to repair the damaged areas themselves without any external aid. There are many different smart coating systems, including ones that are self-healing, conductive, antifouling, antibacterial, and others.^8−11^ Finding a material with a high degree of safety, a long lifespan, and cheap maintenance costs is difficult.^12^ The self-healing process might be a useful substitute because it is not feasible to look for the ideal material. Self-healing corrosion protection coatings have recently been the subject of in-depth research. It is commonly known that these coatings may increase public safety, save maintenance costs, and extend the lifetime of the metal.^13,14^ Based on the coating components or formulation, smart coatings can be organic,^5,15,16^ inorganic,^17−19^ or hybrid (a combination of organic and inorganic components).^20,21^ Self-healing is often maintained by using a self-healing agent like dicyclopentadiene,^22^ linseed oil,^23^ tung oil,^24^ and linalyl acetate.^25^ There are different formulations of the shell that contains the self-healing agent including urea-formaldehyde microcapsules (UFMCS)^26−28^ or multilayered urea-formaldehyde microcapsules (MLUFMCs),^28^ melamine urea-formaldehyde (MUF),^27^ polyurethane (PU),^29^ etc. Moreover, self-healing can be carried out using several corrosion inhibitors, which act as a protective species and inhibit corrosion by sensing pH, pressure, or temperature changes.^30−32^ To control the inhibitor release and maintain it in the coating, different kinds of nanocontainers can be loaded with the inhibitor, which provides a resistive structure against the corrosive media.^33−35^ The nanocontainers can be presented in different forms based on their structure.^25,35−40^ Layered double hydroxide (LDH) is categorized as an inorganic anionic clay nanomaterial composed of nanosheets with a positive charge and anionic and solvent molecules established between them.^41^ The general structural formula of the layered double hydroxide is [M_1–*x*_^2+^M_*x*_^3+^ (OH)_2_]^*x*+^(A^*n*–^) _*x*/*n*_·mH_2_O that comprises divalent metal cations (M^2+^) such as Zn^+2^, CO^+2^, Cu^+2,^, and Fe^+2^, trivalent metal cations (M^3+^) such as Cr^+3^, Al^+3^, and Fe^+3^, and the interlayer charge compensating anion (A^*n*–^), which can be substituted with another anion. Numerous investigations were conducted using the LDH nanosheets as nano reservoirs for corrosion protection purposes. Li et al. designed a triple-layer composite coating consisting of a Ni (ENP) underlayer, a Ni–Al layered double hydroxide (LDH) middle layer, and silane (PFDTMS) deposited into Mg alloy.^42^ The developed triple coating showed long-term corrosion resistance when exposed to a 3.5wt % NaCl solution with a superhydrophobic property against water and several typical drinks in daily life. Hu et al. developed highly corrosion-resistant MgAl-LDH/MBT composite coatings through the direct addition of the MBT inhibitor, which showed good corrosion protection to Mg alloy when exposed to NaCl solution.^43^ Several studies have investigated those multileveled coating systems as an alternative to traditional single-layered coating systems and found that they exhibit superior corrosion resistance.^44^ Due to fewer defects and pores in the coatings in a multilayered coating system, the anti-corrosion property was improved.^45^ Hassanein et al. developed a multilayered epoxy coating (DLPCs) with halloysite nanotubes loaded with benzotriazole reinforced in the epoxy coating as a prelayer and melamine urea formaldehyde microcapsules reinforced in the epoxy coating as a top layer.^46^ DLPCs showed a corrosion resistance of 4.19 GΩ cm^2^ after 9 days of immersion in NaCl corrosive solution. Habib et al. designed a multilayered epoxy coating using zirconia nanoparticles as nanocarriers, which were loaded separately with imidazole and PEI to be applied as a prelayer and a top layer of epoxy in the steel, respectively.^47^ The developed coating showed a corrosion resistance of 1.00 GΩ cm^2^ within 7 days of immersion in NaCl. Furthermore, the presented work is a continuation of our published work^48^ to examine the anti-corrosion behavior of the modified urea-formaldehyde microcapsules encapsulated with linalyl acetate when incorporated into a multilayered system and the compatibility of this type of capsule with the system formulations. Compared to the reported similar multilayered systems, the developed system provides higher corrosion resistance and a prolonged lifetime of the coating. An epoxy coating system with a double layer containing a first layer (pre-layer) of epoxy intercalated with Zn/Al LDH loaded with a DOD inhibitor and referred to as LDH-EP and a second layer (top layer) of urea-formaldehyde microcapsules encapsulated with linalyl acetate (UFMLA COAT) intercalated in the epoxy matrix has been developed in the current work. When compared to monolayers, the unique double-layered smart coating system (DL-EP) proposed herein exhibits better corrosion inhibition and self-healing capabilities, making it appropriate for several industrial applications.

## 2 Experimental Section

### Materials

The chemicals used in the synthesis of the microcapsules and in preparing the coatings are mentioned in detail in our previous work.^48^ Zinc nitrate hexahydrate and aluminum nitrate nonahydrate, NaNO_3,_ and NaOH were used to prepare the Zn/Al LDH nanosheets. Dodecylamine (a corrosion inhibitor) and sodium chloride were used to prepare the corrosive media (3.5 wt % with pH 6.7). The used chemicals were purchased from Sigma Aldrich (UK), except the plain carbon steel substrates, which were obtained from a local source with the composition of 0.21% C, 0.30% P, 0.04% S, 0.20% Cu, and 99.18% Fe and a thickness of 1.25 mm.

### Synthesis of UFMCs

Our previous work presented the detailed steps of the urea-formaldehyde microcapsule synthesis encapsulated with linalyl acetate, a self-healing agent.^48^

### Synthesis of Zn/Al LDH

**Figure 1:**
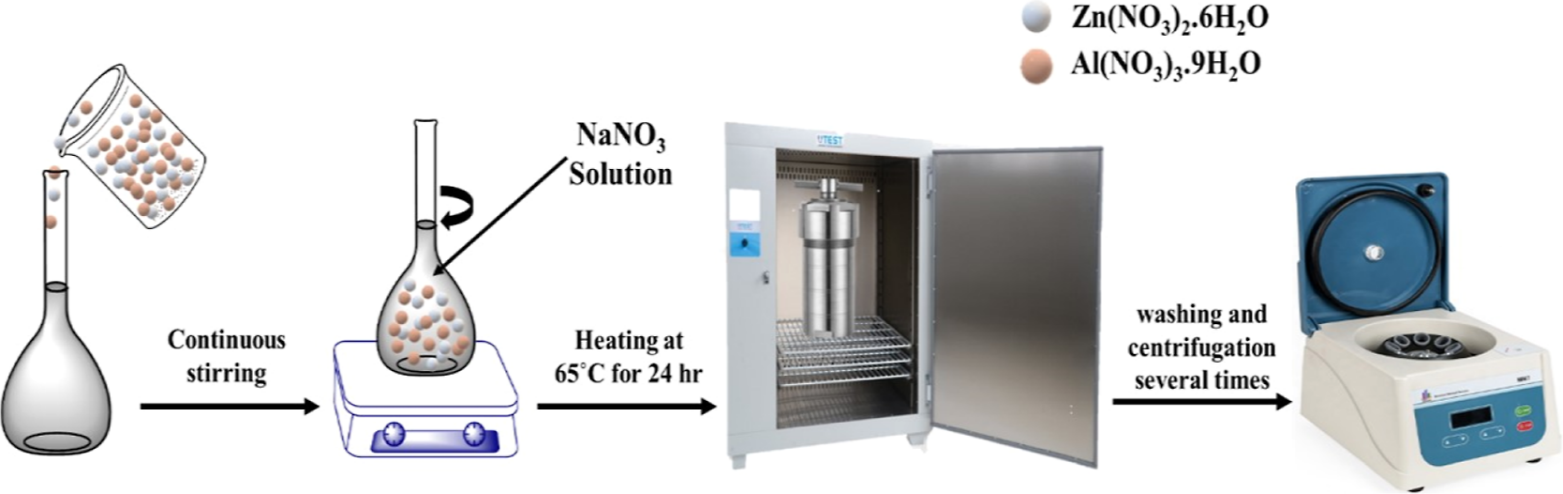
Preparation process of Zn/Al LDH.

The Zn/Al LDH was synthesized by the hydrothermal crystallization method. 100 mL of 1.5 M NaNO_3_ was combined with 50 mL of 0.5 M zinc nitrate and 0.25 M aluminum nitrate solution. The pH of the solution was then progressively adjusted to ∼10 by the addition of 2 M NaOH solution, while it was held at room temperature and continually stirred. The resultant slurry was hydrothermally treated for 24 h at 65 °C to create LDH with high crystallinity. The produced emulsion was repeatedly cleaned and centrifuged to collect the final product. Figure 1 explains the preparation process of Zn/Al LDH. The inhibitor-loading procedure was carried out using the vacuum cycling method, which involved the preparation of a saturated solution of the inhibitor by the continuous stirring of 0.1 M of dodecylamine at room temperature for 2 h. Then, Zn/Al was added to the inhibitor solution and stirred at room temperature for 30 min. After that, the Zn/Al LDH/drug suspension was sonicated for 5 min and placed in a vacuum furnace for 24 h to allow the reduction in the pressure condition to 0.01 atm. Then, the slurry was collected by centrifugation, washed three times with water to remove the unbound molecules, and dried under vacuum at room temperature.

### Preparation of Substrates and Coatings

**Figure 2:**
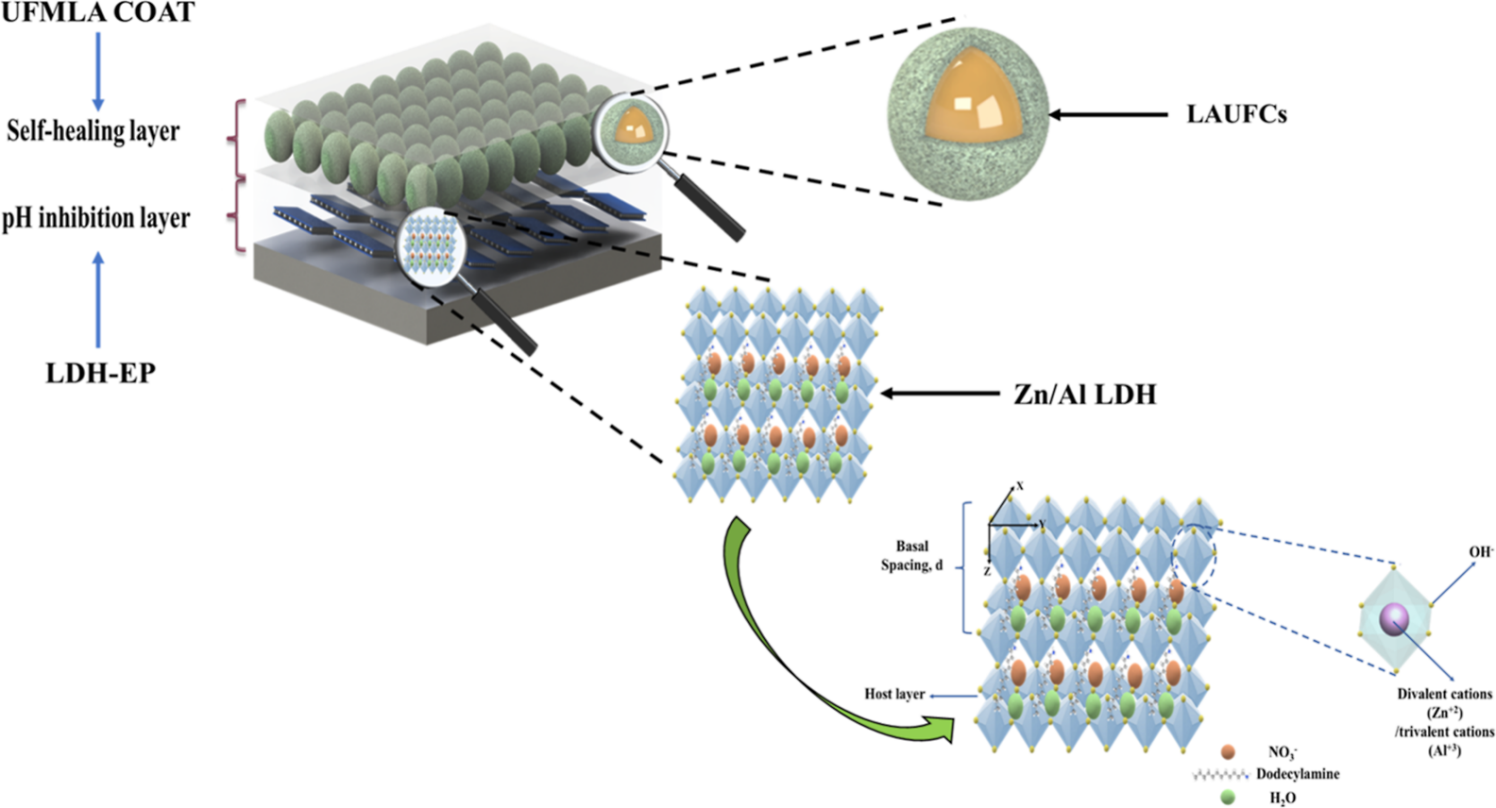
Schematic representation of the proposed double-layer system (DL-EP).

Different coatings were developed for comparison and analysis purposes, which include blank epoxy coatings (reference coatings), a pre-layer containing 3 wt % Zn/Al LDH loaded with DOD referred to as LDH-EP, a top layer of 5 wt % LAUFCs encapsulated with linalyl acetate (UFMLA COAT), and a double layer coating (LDH-EP/UFMLA COAT) referred to as DL-EP, which is presented in Figure 2. The weight percentage of microcapsules has been chosen according to our previously reported study.^48^ To produce the coatings, the epoxy was mixed individually with the LAUFCs, LDH, and hardener (in a ratio of 5 epoxy/1 hardener). Before coating the substrate, the mixture was left in a sonication machine for 5 min to confirm that the LAUFCs and LDH were evenly distributed throughout the epoxy and hardener mixture and that any air bubbles had been eliminated. After that, the epoxy and nanocontainer mixture was stirred under a vacuum at a temperature of 60 °C for 1 h for complete mixing. Then, the mixture was cooled down before adding the hardener, and it was stirred for 15 min. The pre-layer (LDH-EP) was applied to the steel substrate using the doctor blade technique and cured at 25 °C in the air for 48 h. Then, the top layer (UFMLA COAT) was applied to the LDH-EP by the same technique.

### Characterization

The morphology and composition of the synthesized LAUFCs and Zn/Al LDH were studied by a field emission scanning and transmission electron microscope (FE-SEM-TEM-Nova Nano-450) coupled with an EDX analyzer. The thermal stability for the inhibitor, loaded and unloaded Zn/Al LDH, was investigated by thermogravimetric analysis (TGA, 4000, Perkin Elmer, USA). The test was conducted in a temperature range of 40–600 °C with an applied heating rate of 20 °C/min. A Fourier-transform infrared spectroscopy (FTIR) test was conducted on LAUFCs), Zn/Al LDH loaded with DOD, and the developed coatings (reference, LDH-EP, UFMLA COAT, and DL-EP) applying the FTIR Frontier instrument (Frontier-MIR, Perkin Elmer, USA) The main concept of the test is to evaluate the ability of each bond in the tested substance to absorb infrared radiation at a specific absorption frequency range, which acts as a fingerprint for each bond.^13^ The FTIR analysis was carried out in the range of 4000 to 500 cm^–1^. Electrochemical impedance spectroscopy (EIS) was performed using a Gamry device (Reference 3000, Potentiostat/Galvanostat, USA) at room temperature to examine the corrosion resistance of the developed coatings when subjected to controlled mechanical damage in 3.5 wt % NaCl solution. During the test, the developed coatings and a graphite rod were used as the working and counter electrodes, respectively, while the reference electrode was a narrow tube containing KCl aqueous solution. The Gamry cell was filled with 3.5 wt % NaCl solution in which the corrosion test was carried out at room temperature in the frequency range of 0.01 to 100,000 Hz with an AC voltage of 10 mV. BET analysis was carried out for Zn/Al LDH particles to measure the specific surface area of the sample and predict the loading of the inhibitor to the Zn/Al LDH particles, which are directly related to the specific surface area. Furthermore, XRD (X-ray diffraction) analysis was carried out to evaluate the structural properties. XRD was performed using the PW 1800 Philips X-ray spectrometer with Cu-Kα radiation (λ = 1.54060 A) on the synthesized pigment over the 2θ range from 0 to 60° at the rate of 2.5 °C/min. Moreover, XPS (X-ray photoelectron spectroscopy) was performed to determine the composition of the main elements contained on the surface of the carbon steel substrate when exposed to EIS.

## Results and Discussion

### Morphological Analysis

**Figure 3:**
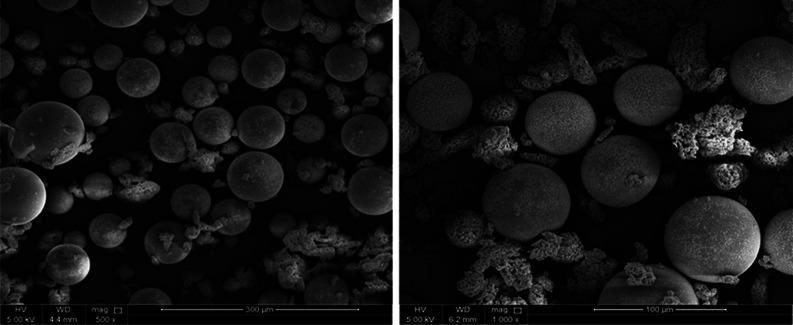
Surface morphology of urea-formaldehyde microcapsules loaded with linalyl acetate LAUFCs.

**Figure 4:**
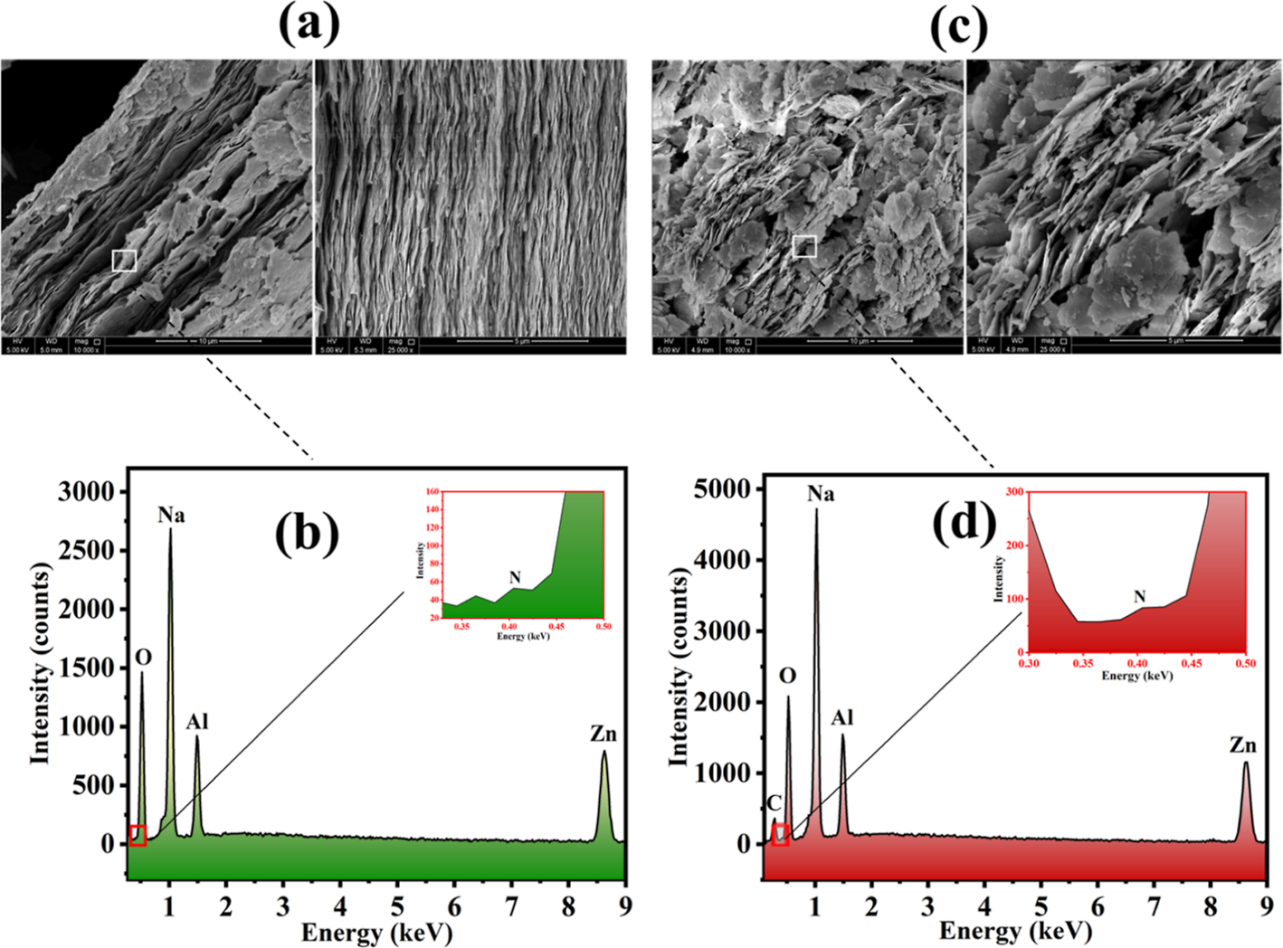
(a,c) SEM of unloaded and loaded Zn/Al LDH, (b,d) EDX of unloaded and loaded Zn/Al LDH.

**Figure 5:**
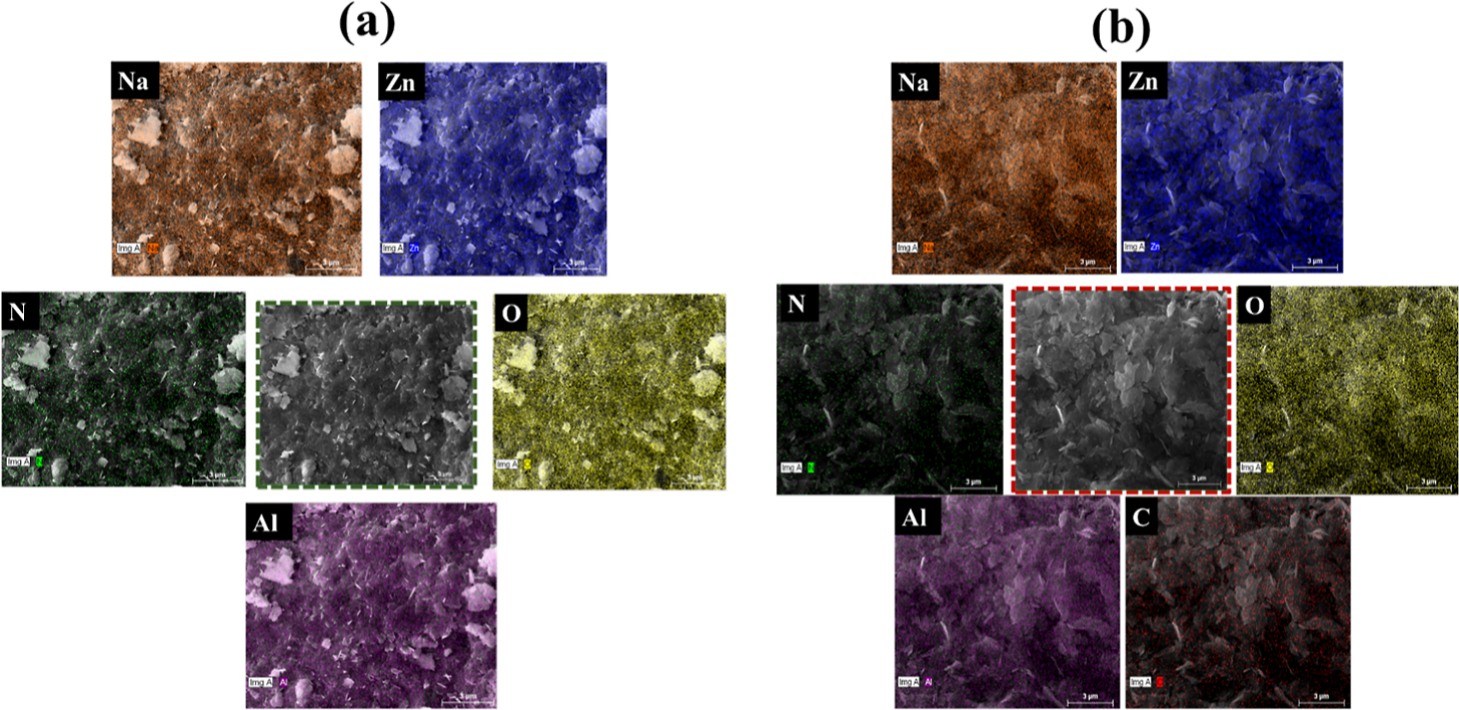
(a,b) Elemental mapping of unloaded and loaded Zn/Al LDH, respectively.

**Figure 6:**
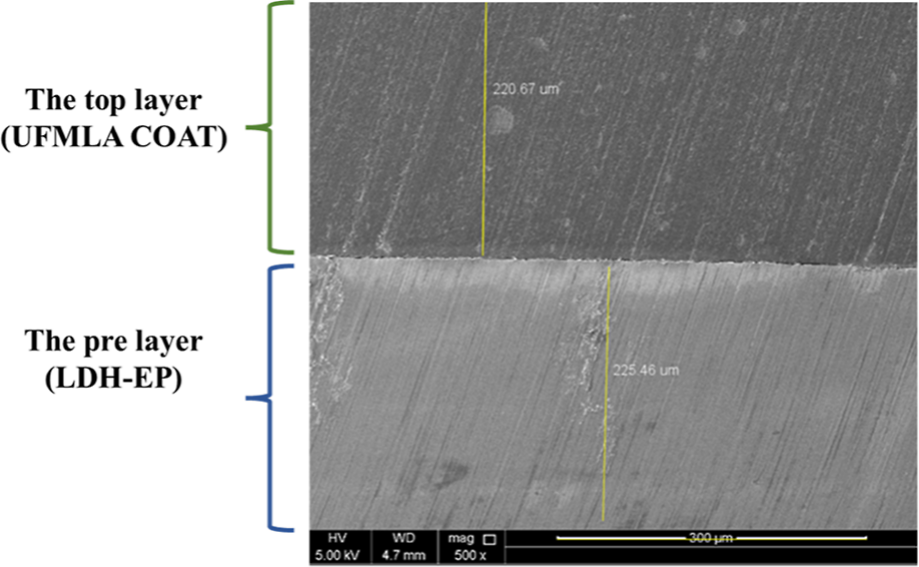
Cross-sectional microstructure observation on DL-EP.

SEM, TEM, and EDX analyses have been carried out for the unloaded and loaded Zn/Al LDH, while for the morphological structure, the detailed discussion of the SEM and EDX analyses of the LAUFCs has been reported in our previous work^48^ and the surface morphology of LAUFCs is shown in Figure 3. Furthermore, the study highlighted that the majority (∼ 70%) of the LAUFCs have a size of 4–125 μm. Figure 4 shows the SEM micrographs and the EDX of the LDH loaded with DOD and unloaded LDH. The Zn/Al LDH sheets in Figure 4a,c have a smooth texture, which is a sign of the material’s high crystallinity. Furthermore, these kinds of materials have a high surface area because of the smooth structure of Zn/Al LDH sheets.^49^ The superposition of several sheets gives the crystals a comparable morphological structure and a well-organized pattern. Moreover, the fundamental reason for the slight morphological variation of the layers is the crystallinity reduction, which was specifically observed by XRD analysis in the loaded and unloaded Zn/Al LDH. Additionally, the loaded Zn/Al LDH’s unstructured behavior and observed spaces are shown in Figure 4c. This may be related to the loading of dodecylamine (DOD) between the Zn/Al LDH. The elemental mapping and EDX analyses of the loaded and unloaded Zn/Al LDH, respectively, are shown in Figures 4b,d and 5. Due to the loading of the inhibitor (DOD-dodecylamine), which includes both nitrogen and carbon, a rise in the nitrogen weight percent and presence of carbon in the loaded LDH can be seen. Because the loaded inhibitor does not include oxygen, as can be observed, the oxygen amount remained unchanged after the loading. The presence of desired elements confirms the purity and absence of any unwanted reaction during the loading of DOD into Zn/Al LDH. Furthermore, the cross-sectional microstructure observation of the DL-EP is provided in Figure 6 which shows the presence of two different epoxy layers with good adhesion with the thickness map of each layer.

**Figure 7:**
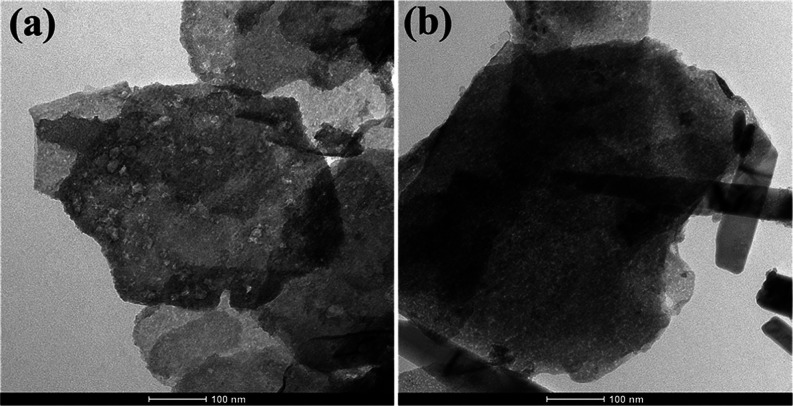
TEM micrographs of (a) loaded Zn/Al LDH (b) unloaded Zn/Al LDH.

Figure 7a,b displays TEM images for loaded and unloaded Zn/Al LDH, respectively. The prepared Zn/Al LDH is made up of hexagonal platelets with a plate-like particle and minor cracks at their edges, as shown by the transmission electron microscopy (TEM) micrographs. Some of the presented sheets also display a vertical crossing at the hexagonal sides. Additionally, it was noted that the unloaded Zn/Al LDH, shown in Figure 7a, had an aspherical form. The type and quantity of the interlayered anions have a major role in how differently the sheets differ in size and shape.^50^

### Thermal Stability

**Figure 8:**
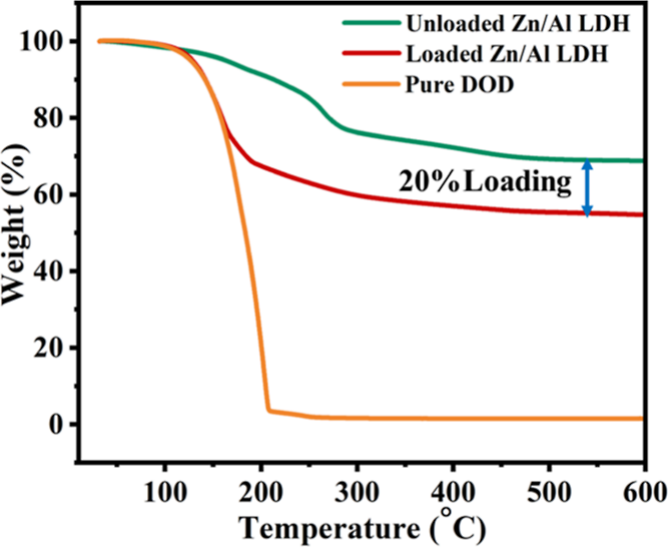
TGA patterns of unloaded and loaded Zn/Al LDH and pure DOD.

Figure 8 represents the TGA patterns of unloaded LDH, loaded Zn/Al LDH, pure DOD, LAUFCs, pure LA, and coatings. As shown in Figure 8, the unloaded Zn/Al LDH decomposes during three primary phases. Since the moisture released from the LDH surface and interlayer evaporated with the rise in temperature, there is an 8–10% weight loss in the first stage, which occurred in the temperature range of 70 to 180 °C. The removal of water absorbed on the LDH surface and the interlayer produces dryness of the LDH layers, generating a weight loss of 10–13% in the second stage at temperatures ranging from 180 to 280 °C accompanied by a heat flow.^49^ In the third stage, a full decay has been carried out in the temperature range of 280–500 °C of the LDH, presenting a 12% weight loss because of the nitrite ion and hydroxyl group removal from the LDH interlayer.^49,50^ As the temperature reaches 550 °C, the TGA profile of unloaded Zn/Al LDH shows a total weight loss of ∼30%. As shown in Figure 8, the TGA profile of the loaded LDH shows a degradation of the material’s mass by 42% through the temperature range of 100–300 °C, which may be attributed to the degradation of the inhibitor loaded into the LDH. The percentage of the inhibitor loading into the LDH is ∼20%, which can be observed from the difference in weight loss between unloaded and loaded LDH after their full degradation at 550 °C. Moreover, as is also evident from Figure 8, the complete degeneration of the loaded LDH at 260–270 °C is an indication of the effective loading of the inhibitor, which has a boiling point of 259 °C.^51^ The TGA of pure linalyl acetate (LA) and urea-formaldehyde microcapsules modified with linalyl acetate (LAUFCs) was illustrated in our previously published work.^48^

### FTIR Analysis

**Figure 9:**
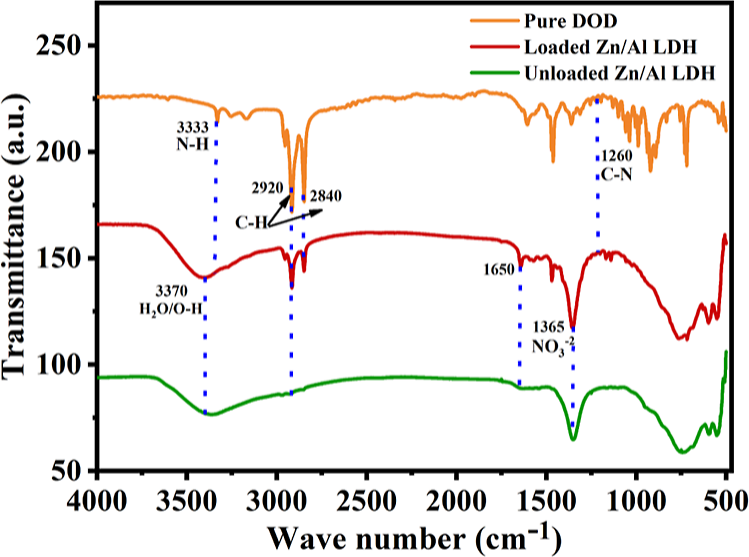
FTIR patterns of pure DOD, unloaded Zn/Al LDH, and loaded Zn/Al LDH.

The usage of the FTIR is mainly to assure the loading of DOD in the Zn/Al LDH interlayers and the effective encapsulation of LA in the shells of UFMCs. The FTIR spectra of dodecylamine, unloaded, and loaded LDH are shown in Figure 9. A wide peak at 3331 cm^–1^ due to the N–H bond can be observed on the FTIR spectrum of the pure dodecylamine, showing a broad peak at 3333 cm^–1^ for the N–H bond presence. Furthermore, peaks with high intensities can be seen at 2920 and 2840 cm^–1^, which is attributed to the long C–H chain contained in the DOD structure. In addition, the presence of a C–N bond in the DOD can be confirmed with the peak at 1260 cm^–1^.^52,53^ The FTIR pattern of the unloaded Zn/Al LDH presents a broad peak at 3370 cm^–1^, which is attributed to the stretching of the O–H bond in the OH groups and water. The H_2_O bending vibration of the interlayer water can be seen in the peak at 1650 cm^–1^. Moreover, the dominant peak at 1365 cm^–1^ could be due to the anti-symmetric stretching mode of the NO_3_^–^, which intercalated between the Zn/Al LDH interlayers_._ In the FTIR pattern of loaded Zn/Al LDH, the sharp peaks at 3370, 2920, 2840, 1650, and 1365 cm^–1^ show the effective loading of DOD into the Zn/Al LDH. The FTIR of the pure linalyl acetate and the LAUFCs have been discussed in our previous work.^48^ The presented findings are in agreement with the literature.^25,50,54,55^

### BET Analysis

**Figure 10:**
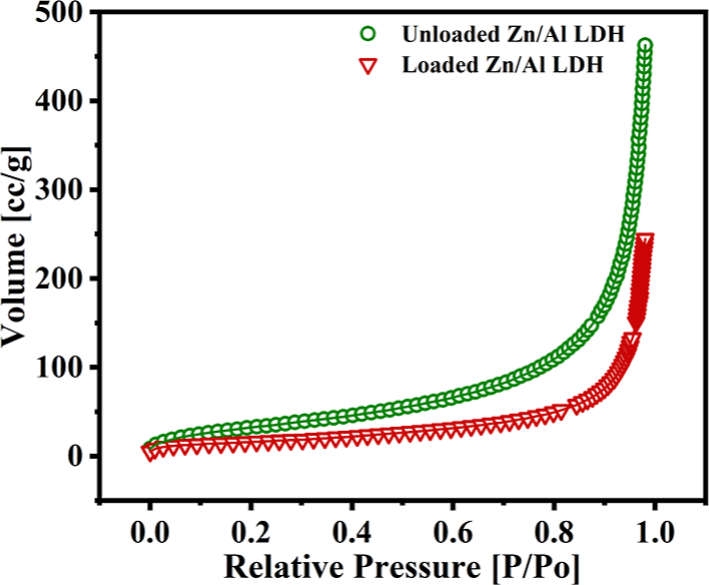
N_2_ adsorption isotherms of the unloaded and loaded Zn/Al LDH.

BET analysis was conducted for the loaded and unloaded Zn/Al LDH to check that the DOD inhibitor was successfully loaded between the LDH interlayers. Because internal LDH surfaces are difficult to identify, standard BET has been used to represent the LDH-specific surface area, which varies from 20 to 100 m^2^.g^–1^.^56^Figure 10 shows the nitrogen adsorption isotherms for the unloaded and loaded LDH. It can be seen that the adsorption isotherms of the synthesized LDH (loaded and unloaded) are following type III (as per the IUPAC categorization). Adsorption occurs in four phases as the gas pressure rises. At the low-pressure range, the nitrogen starts to be adsorbed at isolated spots on the sample surface. As the pressure rises, the absorbed gas molecules begin to cover the sample pores and create a monolayer. The more increase in the nitrogen gas pressure induces the accumulation of gas on the sample surface, which clogs its holes. The specific surface area and the pore volume of the loaded Zn/Al LDH sample were 69.92 m^2^ g^–1^ and 0.385 cc/g, respectively, which are less than the unloaded LDH sample, which is equal to 155.43 m^2^ g^–1^ and 0.735 cc/g, respectively. The reduction in the surface area and pore volume of the loaded sample shows the successful loading of the DOD into the prepared LDH. The inhibitor loading lowers the inner space of the pores, reducing the absorption of nitrogen into the pores. The reported findings are consistent with those reported previously in the literature.^56^

### X-ray Diffraction (XRD)

**Figure 11:**
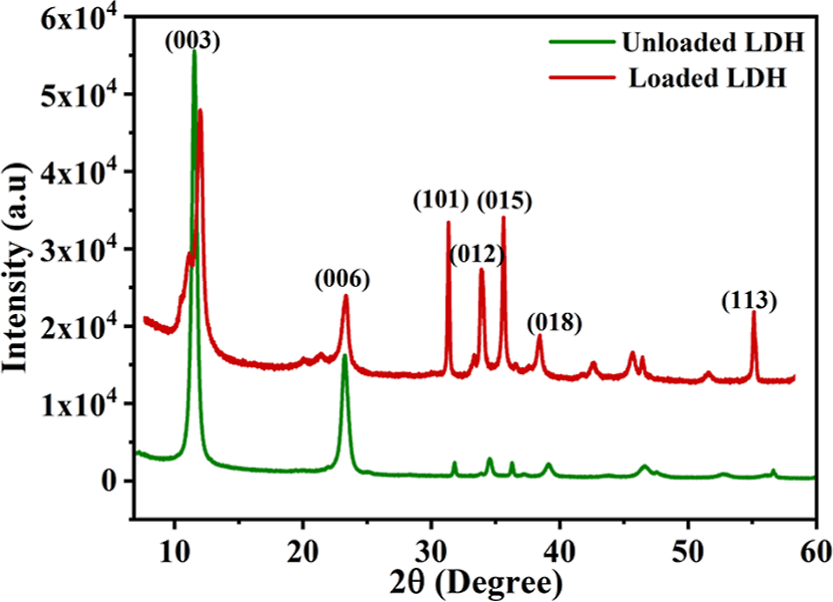
XRD pattern of unloaded and loaded and Zn/Al LDH.

Figure 11 presents the XRD patterns of the loaded and unloaded LDH. The featured crystallinity of unloaded and loaded LDH is demonstrated by reflections of layered double hydroxides intercalated with NO_3–_. The strong basal peaks at 11.5 and 23.5° correspond to reflections of the Zn/Al LDH phase with the R-®3m rhombohedral symmetry (003) and (006), respectively. The peaks at the (003) and (006) planes are the most dominating, acting as a fingerprint verifying Zn/Al LDH’s R̅3m rhombohedral symmetry.^50^ The (003) reflection determines the effective intercalation of nitrate anions inside Zn/Al LDH layers. The shown peaks present the typical structure of Zn/Al LDH. Because the spacing between the basal plans is determined by the size of the primarily intercalating anions, Zn/Al LDH is composed of a single domain intercalated by nitrate anions. Furthermore, the XRD data demonstrate that LDH was synthesized in a pure phase utilizing the hydrothermal crystallization process. As a result, there are no reflections of other phases or contaminants in the XRD pattern. The unloaded LDH XRD spectrum is following the reference code (ICDD:98-018-4799). However, the loaded LDH XRD spectrum follows the reference codes (ICDD:98-018-4799 and ICDD:98-018-2055).

### Electrochemical Impedance Spectroscopy (EIS)

**Figure 12:**
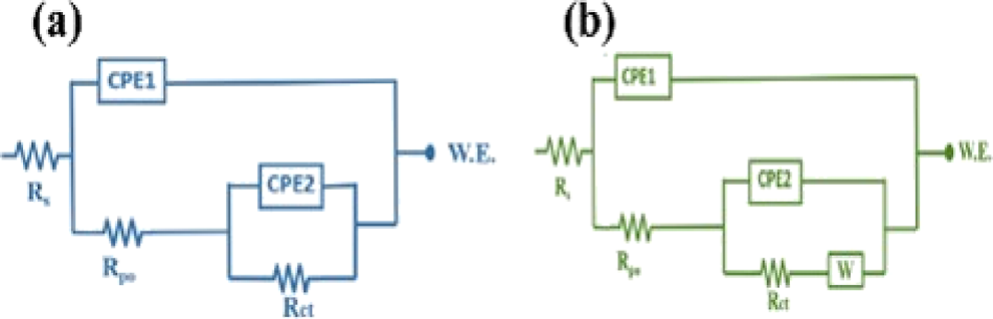
Electrical circuits used for EIS fitting for (a) LDH-EP and (b) DL-EP.

EIS analysis has been conducted using a continuous immersion in 3.5 wt % NaCl solution at room temperature for 60 days to assess the anti-corrosion capability of the produced LDH-EP and DL-EP. Figure 12 depicts the two-time constant electrical circuits utilized in the fitting of EIS experimental data of the two produced coatings to quantify key impedance properties. Rs, Rpo, Rct, CPE1, CPE2, and W are the electrolyte resistance, coating pore resistance, charge transfer resistance at the metal-coating interface, constant phase elements, and the Warburg diffusion constant, as the order given, in the applied equivalent electrical circuit. It is critical to incorporate the constant phase element admittance instead of the capacitance, as the developed coating does not act as an ideal capacitor (*n* = 1). Hence, there is a need to represent surface roughness and non-homogeneity caused by the adsorption of certain species on the metal surface, forming a passive film.^47^ Furthermore, it accommodates non-uniform current distribution throughout the coated surface.^57^

**Figure 13:**
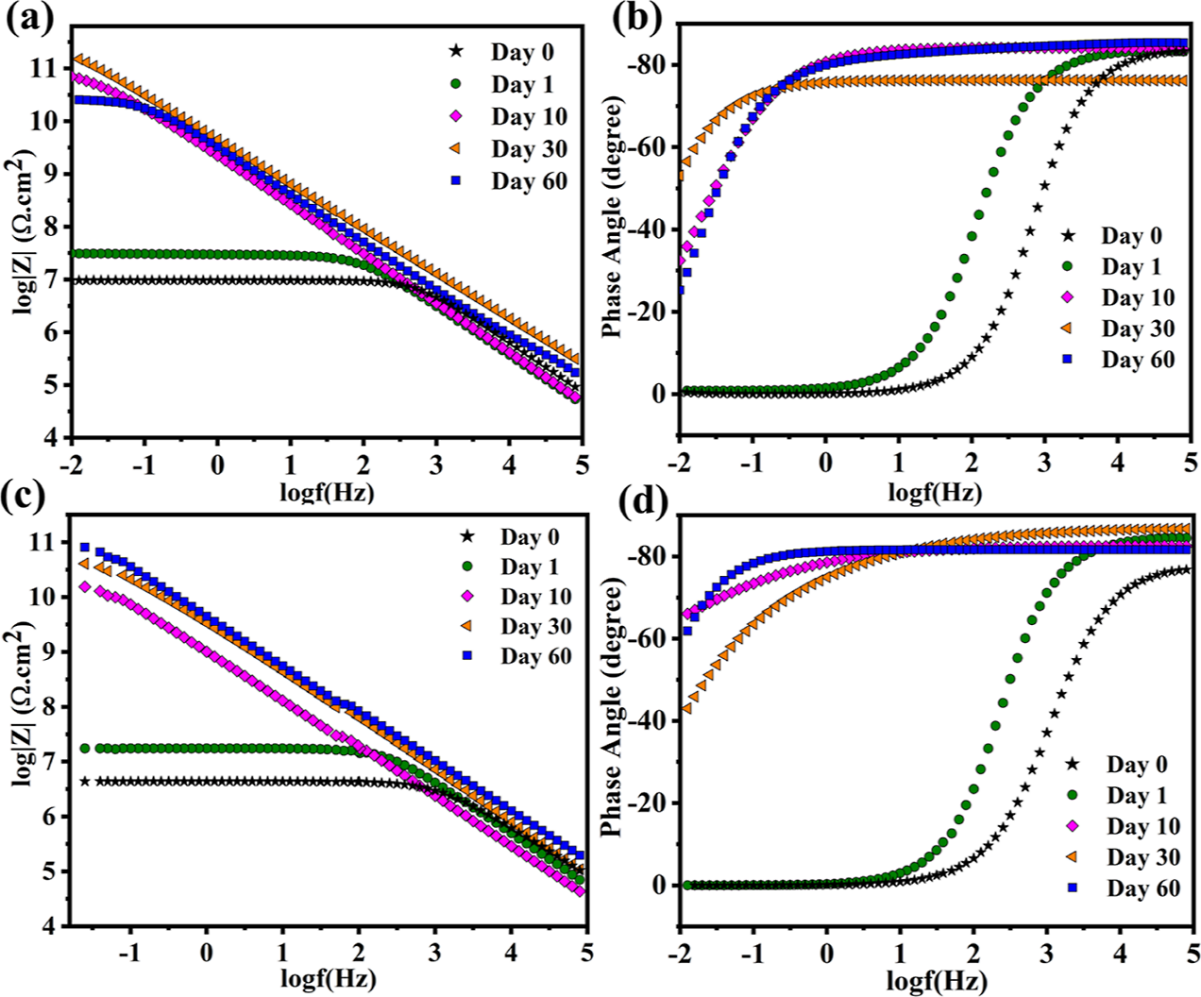
LDH-EP and DL-EP (a,c); EIS Bode graphs; and (b,d) phase angle graphs, respectively.

The Bode graph and the phase angle of single-layer epoxy coating reinforced with loaded LDH (LDH-EP) are shown in Figure 13a,b. The coated samples’ Bode plots were displayed on days 0, 1, 10, 30, and 60 after a consistent scratch was performed on the coating to enable the flow of the electrolyte, which initiates the inhibitor release. The anti-corrosion behavior of the blank epoxy coating and the LAUFC epoxy coating (UFMLA COAT) was studied intensively in our recently published work.^48^ As previously stated, the charge transfer resistance of the blank epoxy coating drops as immersion duration rises, highlighting the corrosion initiation due to the absence of the self-healing or corrosion inhibition effect. Similar behavior is seen in the UFMLA COAT, which exhibits electrolyte solution contact with the steel through the scratched zone of the coatings. Contrary to the UFMLA COAT and the blank epoxy coating, Figure 13a indicates that the charge transfer resistance at the low-frequency range of the LDH-EP increased gradually after 10 days of continuous immersion from 10 M to 75 GΩ cm^2^. This ongoing rise in the impedance value is anticipated to be due to the effective release of the DOD from the zinc-aluminum LDH. Furthermore, the phase angle trend agrees with the Bode graph trend, which shows an increase in the global impedance reaching −60° before progressively falling to −30° (in the low-frequency range) after 60 days of the experiment.

**Figure 14:**
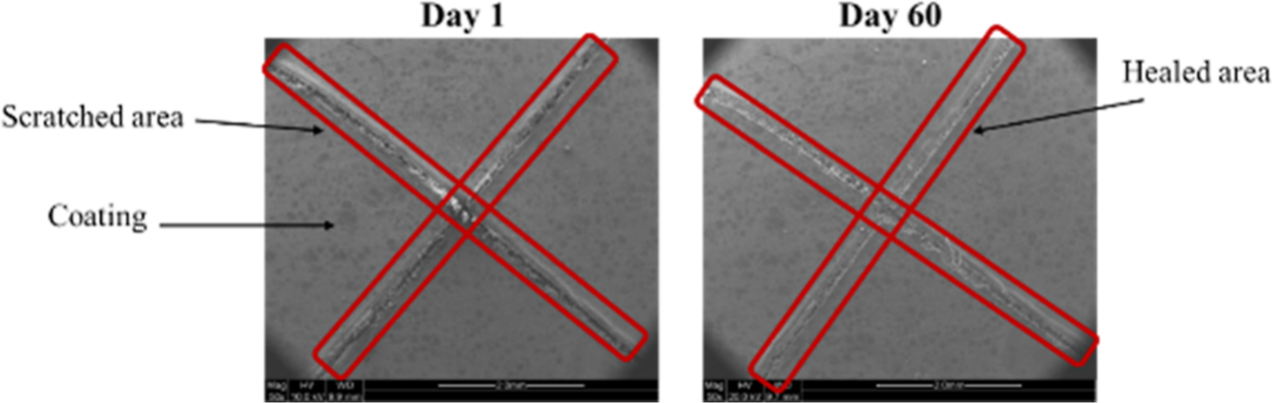
Micrographs of the DL-EP on day 1 and day 60 of the immersion.

The rising trend of the charge transfer resistance at the low-frequency range was corroborated by a widening of the phase angle graphs, as illustrated in Figure 13b, indicating that LDH-EP’s corrosion inhibition capacity has been improved. The charge transfer resistance at the low-frequency range of LDH-EP lowers considerably after 60 days of immersion, reaching 25 GΩ cm^2^ at the end of the experiment. The reason for the significant decline in the LDH-EP corrosion resistance is the absence of the LA (which presented DL-EP) and the corrosion introduction in the damaged areas. The increase in the charge transfer resistance at the low-frequency range for 30 days is proof of the DOD release, which obstructs the corrosion activity for a certain period due to its physiochemical adsorption at the scratched area. However, the DOD release cannot heal the defected zone as the LA can. With further change in the localized pH due to the lineal and continuous contact with the 3.5 wt % NaCl solution, the traces of the electrolyte penetrated the protective film created by the inhibitor, which increased the corrosion activity and caused a decrease in the charge transfer resistance after 60 days of immersion. The withdrawal in the corrosion behavior of the LDH-EP can also be ascribed to the propagation of the corrosive media through the coating micropores reaching the steel/coating interface and causing corrosion products to develop and accelerate the corrosion process. Figure 13c,d presents the Bode and phase angle graphs of DL-EP. The double-layer coating (DL-EP) has an incremental trend in the charge transfer resistance at the low-frequency range throughout the whole experiment, highlighting the self-healing effect in the coating. For instance, the charge transfer resistance at the low-frequency range of the DL-EP rises gradually from 20 MΩ.cm^2^ to 250 GΩ cm^2^ due to LA release, which offers a pre-defending effect for the coating by healing the damaged area on the coating and forming a protective film, which minimizes the area exposed to the corrosive media. In contrast to the LDH-EP that witnessed a 66% decrease in the charge transfer resistance at the low-frequency range in the last 30 days of continuous immersion, the charge transfer resistance at the low-frequency range of DL-EP rises gradually to 250 GΩ cm^2^ that corresponds to a 49% rise in the corrosion resistance in the last 30 days of immersion. The high rise in the charge transfer resistance at the low-frequency range of DL-EP after 60 days of immersion highlights the strong barrier quality and great compatibility of DOD and LA that provide a fully protected anti-corrosive system. Moreover, the reason for the strong inhibitory qualities of the utilized inhibitor DOD can be attributed to the inhibitor’s capacity to raise the resistance of the cathode and anode processes, which impede the cathodic and anodic site activity for corrosion initiation.^52^ Furthermore, the phase angle verifies the incremental charge transfer resistance at the low-frequency range pattern with immersion time, which shows the reaching of the phase angle up to −70°. Figure 14 shows SEM micrographs of the DL-EP, which embodies the self-healing effect at the beginning of the immersion and at the end indicates complete healing of the coating.

To be able to study the self-healing and corrosion inhibition effect quantitatively, an equivalent electrical circuit (illustrated in Figure 12) has been used to fit the experimental data of the EIS to obtain the EIS parameters tabulated in Table 1. First, regarding the LDH-EP, the pore resistance (Rpo) values witnessed an increase through 30 days of continuous immersion in the NaCl electrolyte, indicating the good corrosion resistance and barrier capability of the coating. It can be observed in Table 1 that the Rpo values dropped from 2.085 × 10^–4^° GΩ cm^2^ after 30 days of immersion to 1.836 × 10^–6^ GΩ cm^2^ after 60 days of immersion because of the probability of passive layer degradation induced by electrolyte entry through the repaired region. Unlike the LDH-EP, DL-EP has continuously risen in the Rpo values through the experiment reaching 373.8 GΩ cm^2^, which corresponds to a 99.87% rise as compared with the Rpo value of the LDH-EP, which witnessed a decreasing pattern after 60 days of the experiment. The constant rise in the Rpo values verifies a successful uniform release of the inhibitor and the self-healing agent, which aids in the healing of the coating defect. The higher improvement in the DL-EP demonstrates the multilayer coating’s capacity to provide an additional barrier property with ongoing recovery. The Rpo values of the DL-EP and LDH-EP present a gradually increasing trend over 30 days of the experiment. However, after 60 days of immersion, the Rpo values witnessed a steady increase in the case of DL-EP while decreasing in LDH-EP. On the other hand, Rct values for DL-EP and LDH-EP exhibit comparable growing and declining tendencies, with larger values for DL-EP reflecting its lower interfacial activity relative to LDH-EP. Furthermore, CPE1 and CPE2 exhibit a decremental trend, indicating good capacitive behavior and strong protective capabilities of the coatings.

**Table 1:** Electrochemical Parameters Obtained from EIS Fitting of LDH-EP and DL-EP

coating type	immersion time (days)	Rpo (GΩ cm^2^)	CPE1 (s^*n*^ Ω^–1^cm^–2^)	CPE2 (s^*n*^ Ω^–1^cm^–2^)	Rct (GΩ cm^2^)	*n*	*W*	goodness of fit
single-layer coating (LDH-EP)	0	4.155 × 10^–9^	8.00 × 10^–11^	170.9 × 10^–6^	0.664	0.98		5.892 × 10^–3^
	1	2.601 × 10^–8^	7.24 × 10^–11^	5.573 × 10^–6^	6.320	0.0105		33.20 × 10^–3^
	10	5.064 × 10^–8^	6.28 × 10^–11^	38.08 × 10^–9^	26.210	0.289		12.26 × 10^–3^
	30	2.085 × 10^–4^	5.27 × 10^–11^	34.25 × 10^–12^	532.800	0.256		53.78 × 10^–3^
	60	1.836 × 10^–6^	2.51 × 10^–11^	12.91 × 10^–12^	5.210	0.902		20.73 × 10^–3^
double-layer coating (DL-EP)	0	2.528 × 10^–6^	160.4 × 10^–12^	140.2 × 10^–12^	0.009762	0.950	9.680	58.95 × 10^–3^
	1	2.868 × 10^–4^	83.31 × 10^–12^	72.80 × 10^–12^	0.01735	0.671	6.640	17.56 × 10^–3^
	10	0.00182	63.36 × 10^–12^	57.03 × 10^–12^	97.26	0.965	15.78	9.530 × 10^–3^
	30	0.009693	42.47 × 10^–12^	14.46 × 10^–12^	292.9	0.902	4.380	2.532 × 10^–3^
	60	373.8	8.650 × 10^–12^	71.92 × 10^–18^	58.02 × 105	0.01295	4.860	6.612 × 10^–3^

### Self-Healing and Corrosion Inhibition Mechanism

**Figure 15:**
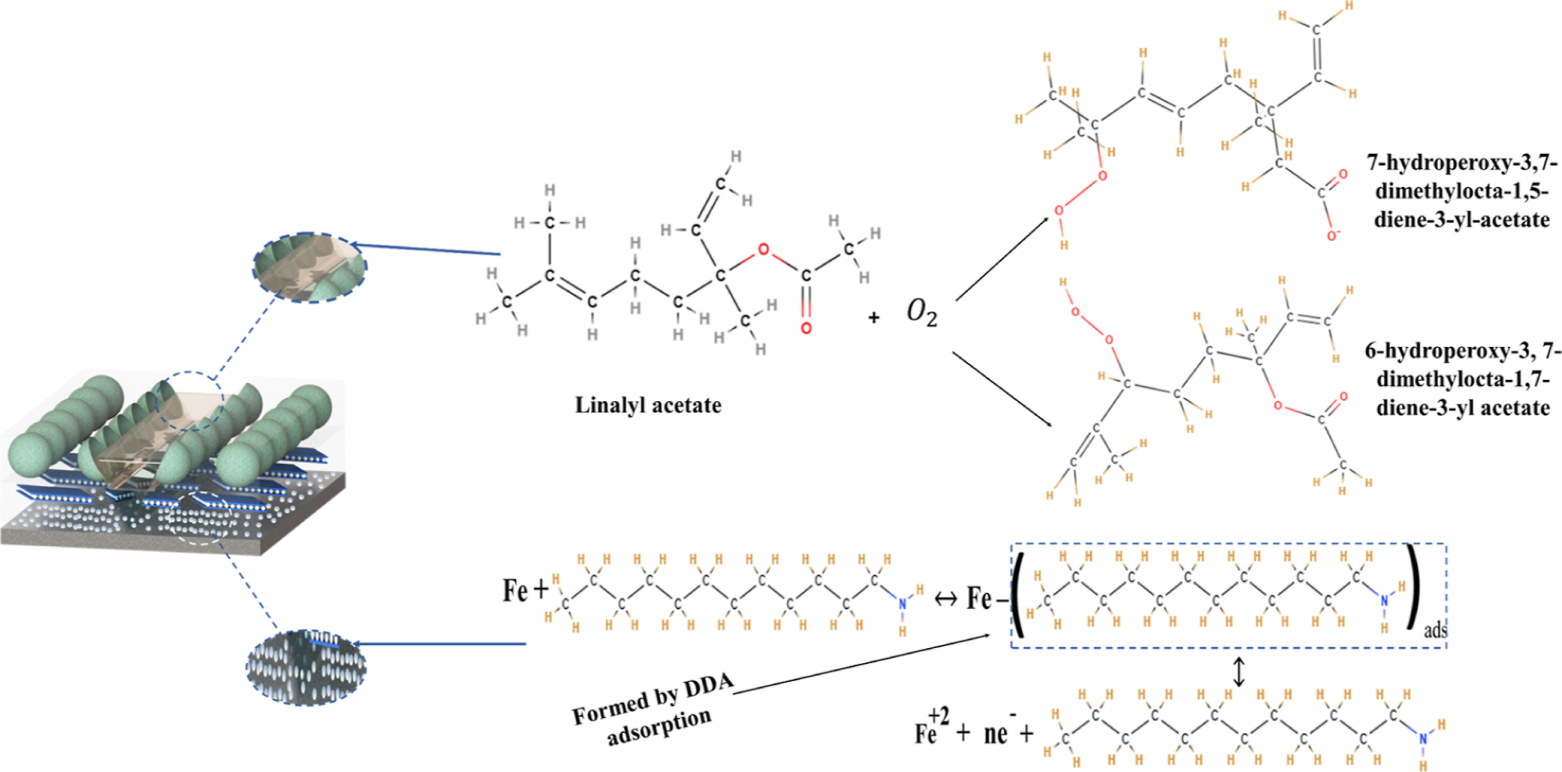
Corrosion protection analysis of DL-EP.

The suggested system (DL-EP) uses two methods of corrosion prevention to offer a completely protected system with high barrier qualities, as illustrated in Figure 14. This protected system is obtained using linalyl acetate and dodecylamine, which are applied as a self-healing agent and a corrosion inhibitor, respectively. As the damaged spots are exposed to the oxygen in the air, the self-healing of LA takes place by the formation of a mixture of two hydroperoxide compounds, which form a thin passive layer on the steel that enhances the corrosion resistance, as discussed in our recent work.^48^ Dodecylamine is a good corrosion inhibitor with a high diffusion barrier due to the van der Waals interactions between the alkyl chain and the very active NH_2_ group. In detail, DOD controls corrosion through two different effects, which are the energy effect and the geometric blocking effect. The geometric blocking effect has been achieved by producing a single layer coating on the substrate surface that reduces the area exposed to the reaction by spontaneous physiochemical adsorption by the presence of the amine group. The energy effect occurs by raising the activation energy of the redox processes happening on the steel surface, which is free of the inhibitor during the surface rate ion process.^53^ The increase in the DOD chain length causes an increase in the DOD concentration, which produces a more uniform, intense layer that results in a higher protective steel surface.^58,59^ The controlled scratch which has been carried out on the epoxy coating surface results in an oxidation–reduction reaction by the immersion into the corrosive media (3.5 wt % NaCl solution), which contains oxygen and water. Metal degeneration takes place (steel oxidation) by the anodic reaction (1). However, the cathodic reaction occurs because the concentration of the inhibitor is low on the steel surface or due to a slow rate of adsorption of the inhibitor, which is considered as a cathodic reaction (2).^60^ Through the direct contact of the NaCl solution with the damaged spots, a change in the localized pH has resulted, which causes the inhibitor release from the LDH nanocontainers. Then, a replacement of the water molecules, which were occupying the steel surface, has taken place because of the released dodecylamine that was adsorbed on the steel surface upon the presented eq 3. As a result, the  intermediate that produces a film of inhibitors has been formed due to the adsorbed dodecylamine on the steel surface, as presented in eq 4. The formed layer/film can be considered as a barrier that impedes the reaching of the chloride corrosive ions to the steel surface. We have noticed a similar release mechanism of DOD from the loaded carriers in the present study as it was reported by us earlier. The detailed release mechanism of DOD has already been explained in our previous publications, in which the release of DOD was evaluated by UV–vis spectroscopy. It was confirmed that the DOD inhibitor had efficient self-release in an acidic medium (pH 2 and 5) with an increase in the release through 72 h. More details on the study could be found through the previous publications carried out by our research group.^35,51^Figure 15 presents the protection analysis offered by the DL-EP by the LA and DOD effects.
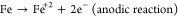
1
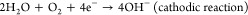
2
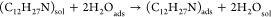
3
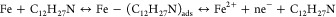
4

### XPS (X-ray Photoelectron Spectroscopy)

**Figure 16:**
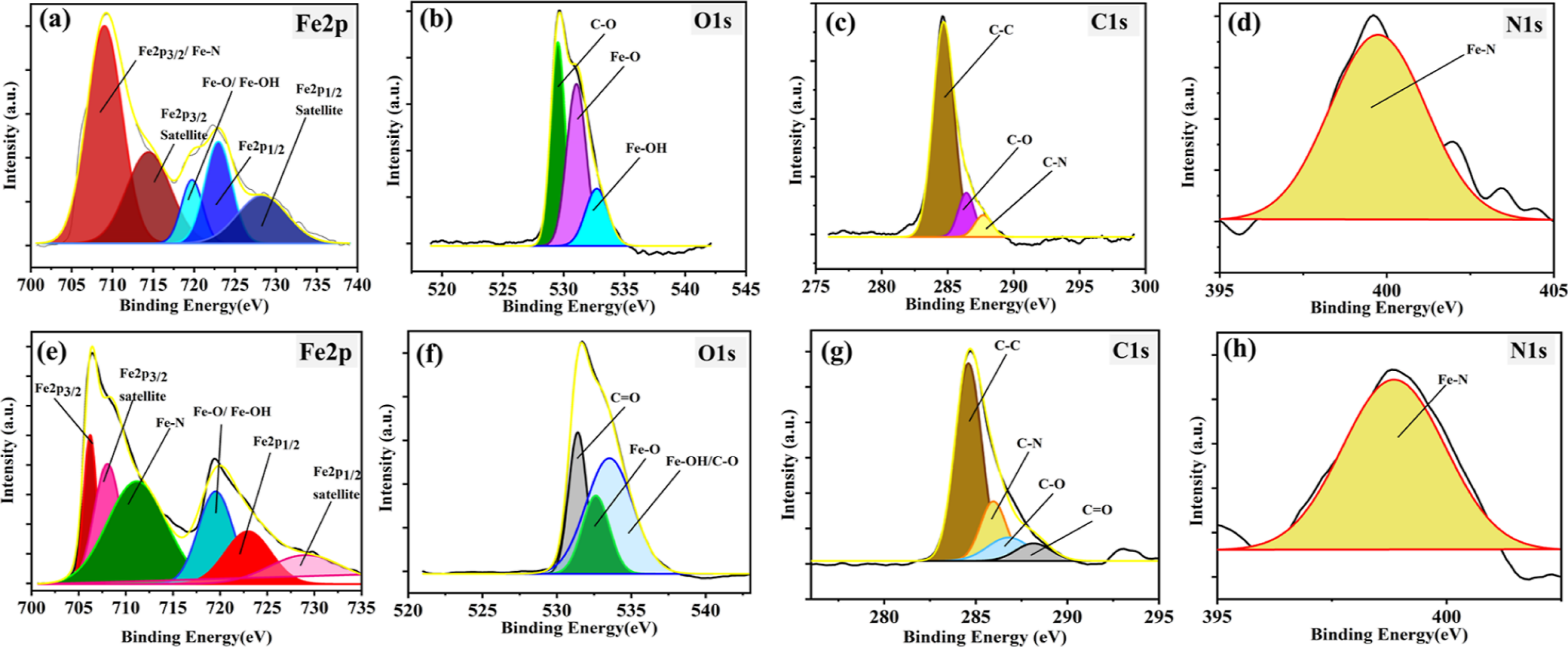
XPS spectrum showing the surface elemental composition of the steel of (a–d) LDH-EP and (e–h) DL-EP.

The surface composition has been obtained for the steel surface to examine the adsorption of the DOD and LA oxidation products on it using the XPS analysis. This was achieved by the LDH-EP and DL-EP epoxy coatings’ removal after 60 days of immersion in the sodium chloride solution. The XPS spectra of Fe, O, C, and N have been shown in Figure 16 for the DL-EP and LDH-EP. Figure 16a,e) shows the XPS spectrum of Fe2p for the LDH-EP and DL-EP, respectively. The peaks at 707 and 720 eV present the metallic form (Fe2p_3/2_) of iron and its satellite, respectively.^61,62^ Moreover, the peaks at 725 and 727 eV present the metallic form (Fe2p_1/2_) of iron and its satellite in the order given.^63^ Furthermore, the 709 eV peak is for the Fe–N bond, which can be attributed to the existence of hydroperoxides (linalyl acetate oxidation products) or the DOD, which can form a bond with Fe.^64^ The peak at 709.5 eV can be for the Fe–O or Fe–OH bonding formed by the reaction of oxygen with the steel surface or the bonding of Fe with the inhibitor; the existence of the hydroperoxides on the steel surface can be seen in the peak at 714 eV.^65,66^ The XPS spectrum of O1s for LDH-EP and DL-EP, respectively, is presented in Figure 16b,f. The Fe–O and Fe–OH bonds which formed because of hydroperoxides and the steel are shown at 531 and 534 eV peaks sequentially. The existence of the C–O bonds composed in the hydroperoxides can be seen in the 530 eV peak.^67^ The C=O bond which is contained only in the hydroperoxides can be seen in the 532 eV peak.^60^ The XPS spectrum of C1s for LDH-EP and DL-EP, respectively, can be seen in Figure 16c,g. The C 1s peak was split into three different peaks, which are C–C, C–O, and C–N at binding energies of 284.8 286.4, and 288 eV, respectively, as shown in Figure 16c.^68,69^ Moreover, the peak at a binding energy of 284.8 eV can be attributed to the C–C/C–H bond between the epoxy, which might remain on the surface of the steel substrate.^70^ On the other hand, the C1s peak in Figure 16g was split into four different peaks. The binding energies of the first three peaks are the same as those in Figure 16c, and the binding energy of the fourth peak was related to the C=O at 288.7 eV.^67^ Additionally, the XPS spectrum of the nitrogen has a single peak at 399.8 eV for the Fe–N bond. This peak confirms the presence of the protective film caused by DOD adsorption.^64,71^ The XPS spectra are proof of the successful adsorption of the LA oxidation products reaching the steel and the capability of the DOD to form a barrier (film) on the steel.

## Conclusions

An efficient, smart double epoxy coating system for corrosion prevention of steel was developed. In this context, Zn/Al LDH loaded with an inhibitor (DOD) and UFMCs loaded with a self-healing agent (LA) were reinforced into an epoxy matrix to form a pre-layer and a top layer, respectively. A comparison of the electrochemical performance conducted on the developed coatings indicates that the double-layer coating (DL-EP) exhibits superior properties when compared to another single-layer coating (LDH-EP) due to the efficient release of an inhibitor and a self-healing agent from the coating layers. The promising anti-corrosion properties of DL-EP make it appropriate to be incorporated into a wide range of applications.
